# Translation and Psychometric Evaluation of the University of Jyvaskyla Active Aging Scale (UJACAS) for Use in Sweden

**DOI:** 10.1007/s10823-024-09496-8

**Published:** 2024-01-22

**Authors:** Frida Nordeström, Björn Slaug, Magnus Zingmark, Marianne Granbom, Taina Rantanen, Susanne Iwarsson

**Affiliations:** 1 Department of Health Sciences, Lund University, Box 117, Lund, 22100 Sweden; 2 Faculty of Sport and Health Sciences, Gerontology Research Centre, University of Jyvaskyla, Jyvaskyla University, Jyvaskyla, Finland

**Keywords:** Healthy ageing, Self-rating scale, Reliability and validity, Outcome measures, Test-retest reliability

## Abstract

The objectives were to translate the University of Jyvaskyla Active Aging Scale (UJACAS) to Swedish, to establish semantic equivalence and evaluate psychometric properties for use among persons 55 years and older in Sweden. The UJACAS contains 17 items to be self-assessed regarding goals, abilities, opportunity, and activity. Psychometric properties content validity, data quality including floor and ceiling effects, test-retest reliability, internal consistency, and construct validity were evaluated with different samples in three phases, using state-of-the-art statistics. After translating and establishing semantic equivalence, content validity was assessed as high. With ICC = 0.88 (95% CI 0.80–0.93) test-retest reliability was moderate. Internal consistency was high (Cronbach alpha = 0.84–0.91), and 84% of the questions reached the cut-off value of 0.3 for corrected item-total correlation. Construct validity hypotheses were confirmed. Results indicate that the UJACAS is reliable and valid for use among persons 55 and older in Sweden.

## Introduction

The World Health Organisation (WHO) has defined active ageing as “…*the process of optimizing opportunities for health, participation and security to enhance quality of life as people age*…” (World Health Organization, 2002, p. 12). From a societal perspective the goal of active ageing has the potential to nurture the adaptation of environments and health-promoting initiatives to better suit the ageing population. Nowadays active ageing is established as a policy strategy to describe the partnership between the citizen and society (Foster & Walker, 2015; Walker, 2002). The European Union (EU) has incorporated the concept in several policy documents, such as the European Commissions’ decision to tackle the ongoing demographic change by raising retirement age and keeping up the numbers of persons still in the workforce as one strategy (European comission, 2012). Although the reason is relevant, there are problems associated with assuming that all older persons would benefit positively and have the possibility to participate in the labour market as they age. Also, the definition used by the WHO and EU for active ageing does not consider the individual aspect, nor those who are more likely to experience significant losses in cognitive and physical potential while ageing (Paúl et al., 2017).

As an outcome measure, the concept has been used on the societal level to rank countries according to indicators such as participation of older people in the workforce and life expectancy (United Nations Economic Commission for Europe) and to measure determinants that are expected to help professionals and researchers to recognize particular profiles that are more at risk or, on the contrary, are more favourable to age actively (Paúl et al., 2017). Even though active ageing measurements between countries and different regions have been found to have common features, they are also culturally specific (Thanakwang et al., 2014).

Rantanen et al. (2019) discussed how a pursuit of active ageing is formed in personal strategies and behaviour to optimize experienced quality of life. To assess active ageing on the individual level the University of Jyvaskyla Active Aging Scale (UJACAS) was developed in Finland (in Finnish) for use in research and practice. Active ageing was defined as “… *the striving for elements of wellbeing through activities relating to a person´s goals, functional capacities, and opportunities*” (Rantanen et al., 2019, p. 1003). With a generic nature of the included items, assessment regardless of functional level was made possible thus shifting the focus to personal aspirations. After the drafting phase, the scale development involved 235 older persons (aged 60–94 years) in a multifaceted process including a pilot study, a feedback study and test-retest study (Rantanen et al., 2019). Evaluation of the psychometric properties demonstrated good test-retest results with a high intraclass correlation coefficient (ICC) for individual subscales and a total scale. Correlations with quality of life, self-rated health, psychological resilience, and life-space mobility supported the construct validity of the scale (Rantanen et al., 2019; Siltanen et al., 2021). To date, UJACAS is available in Finnish, English (GEREC Gerontology Research Center), Turkish (Erbil & Hazer, 2019) and German (unpublished). As psychometric properties are sample dependent, an instrument must be evaluated for every new population (Hobart & Cano, 2009). When introducing an instrument to a population that speaks another language, a translation and semantic equivalence should be addressed before investigating the measurement properties of the translated version of the instrument (Streiner et al., 2015). For a questionnaire to have good measurement properties, a thorough examination of several criteria such as validity, reliability, responsiveness, floor and ceiling effects, and interpretability is warranted (Terwee et al., 2007). Comparisons of results between different populations create a greater understanding of the instrument itself and the concept at target (Davidov et al., 2014). With an increasing number of language versions, comparisons between countries can be made, potentially adding to a European perspective of individual active ageing. Still, in the end the quality of an instrument determines the quality of the results, whether the results provide meaningful information of the population, and what kind of inferences may be possible (Hobart & Cano, 2009).

The objectives of this study were to translate UJACAS to Swedish, to establish semantic equivalence and evaluate psychometric properties (i.e., content validity, data quality including floor and ceiling effects, test-retest reliability, internal consistency and construct validity) for use in Sweden among persons 55 years and older.

## Method

### The UJACAS

**Fig. 1:**
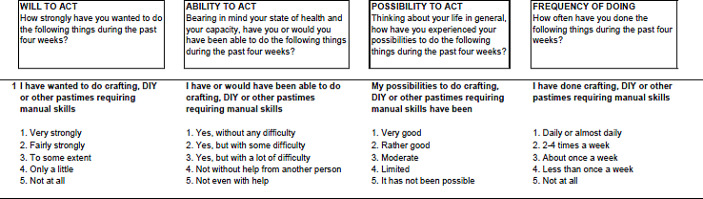
Example from the English version of UJACAS first item (Crafting & DIY), with four subscales and response options

The original UJACAS comprised 17 items to be self-assessed regarding goals, ability, opportunity, and activity, operationalised as four sub-scales (see Fig. 1). The items addressed the following, with abbreviation in parenthesis: Crafting or do-it-yourself (Crafting & DIY), Artistic pursuit (Artistic pursuit), Participating in events (Events), Enjoying nature (Nature), Keeping physically fit (Physically fit), Exercising the mind (Exercise mind), Using computer or pad (Use computer/pad), Supporting or helping others (Support others), Maintaining social relationships (Social relationships), Making new acquaintances (New acquaintances), Taking responsibility in one´s own life (Promote own life), Taking responsibility for societal or communal matters (Public matters), Make one´s days interesting (Interesting days), Maintaining or improving the cosiness of one´s home (Make home cosy), Taking care of appearance (Appearance), Ensuring financial affairs are in order (Financial affairs), and Furthering matters according to faith or world view (Faith or worldview).

Using a five-point Likert scale, participants were asked to rate their answers in the light of the latest four weeks. Response options are worded to suit each item, starting with the alternative representing the highest level of active ageing, in descending order. To calculate a sum score, the response option representing the highest level are assigned 4 points, and the lowest response option is assigned 0. With 17 main items and four subscales, a total of 68 sub-questions adds up to a sum score of 0-272. A higher sum score indicates a higher level of active ageing. A maximum of two missing values are allowed for each subscale, or eight for the total sum score (Rantanen et al., 2019).

### Project Context

This study was performed in the context of the Prospective RELOC-AGE project targeting older adults aged 55 + considering relocation, with active ageing as one of the outcomes. The full scope of RELOC-AGE is available at ClinicalTrials.gov NCT04765696 and in a study protocol (Zingmark et al., 2021).

### Study Design

This study was performed in three phases with different samples. Following the translation of UJACAS from Finnish to Swedish, Phase 1 took place during September to November 2020, with a research team addressing semantic equivalence and a user panel with representatives for the target population addressing content validity. Phase 2 took place during November 2020 to January 2021. Data quality and reliability were assessed in a test-retest involving a convenience sample of people aged 55+. Phase 3 was initiated in April 2021 when the baseline started for the Prospective RELOC-AGE project (Zingmark et al., 2021). In all, the RELOC-AGE data included in this paper was collected from September 2020 until October 2021.

### Participants and Recruitment

In phase 1 the research team addressing semantic equivalence involved some of the co-authors of this paper (FN, MZ, MG, TR, and SI), representing occupational therapy as well as gerontology expertise on junior and senior levels. To assemble the user panel, we used our collected personal networks. The inclusion criteria were age 55+, interest in the concept of active ageing and related research and being a fluent Swedish speaker. Nineteen persons who we knew met these criteria were approached by email and six answered and were included. This was considered an appropriate number of participants for the selected analysis (Polit & Beck, 2006). Women represented half of the user panel (*N* = 3 women), and mean age was 73 years.

For phase 2, the inclusion criteria were age 55 + and a postal address in Sweden. Exclusion criteria were cognitive impairments and/or insufficient language skills to give informed consent and/or participate in telephone interviews, as assessed by first author who has 15 years of experience working with individuals with different cognitive impairments. Also, living in residential care was an exclusion criterion. Information about the study including an invitation to participate was sent to a contact person or board member of senior citizen associations. Of thirteen contacted, eight associations agreed to inform about the study through their newsletters and email communication to members. First author was contacted by 66 persons who wanted to participate, but two chose later to withdraw their participation. Another individual was excluded due to cognitive difficulties apparent during the first interview; despite the interviewer repeating both the purpose of the study and the current item question several times, concerns remained whether the respondent knew which question was being discussed. Thus, 63 persons gave their answers on both occasions, with approximately 13 days in-between. Characteristics of the phase 2 respondents collected: 2/3 were women (*N* = 42), mean age was 75 years (range 61–92) and almost half of the respondents (47%) had a university education of 3 years or more.

For phase 3, participants responding to the Prospective RELOC-AGE web survey were invited to additional data collection by telephone including UJACAS (Zingmark et al., 2021). In all, 1,412 individuals agreed to participate in telephone interviews. Due to administrative reasons, only 1,011 respondents could be invited. Of those, 820 (81%) participated. Half of the included respondents were women (*N* = 415; 50.6%), mean age was 70 years (SD = 7.6). For detailed information of the respondents of phase 3, see Table 1.

**Table 1:** Characteristics of respondents, study phase 3

Characteristic	Phase 3 (*N* = 820)
Sex, women, n (%)	415 (50.6)
Age, mean (range)	70 (55–92)
Educational level^1^, n (%)	
Compulsory school	55 (6.9)
2 years of upper secondary school	68 (8.5)
3 or 4 years of upper secondary school/equivalent	113 (14.0)
University < 3 years	157 (19.5)
University 3 years or more	418 (51.2)
Current occupation^2^, n (%) (several choices possible)	
Employed	201 (24.5)
Self-employed	79 (9.6)
Temporary sick leave exceeding 90 days	2 (0.2)
Parental leave	2 (0.2)
Studying	5 (0.6)
Unemployed	10 (1.2)
Unpaid volunteer work	30 (3.7)
Pensioner	571 (69.7)
Other	59 (7.2)
Type of housing^3^, n (%)	
Apartment	407 (49.8)
House / semi-detached house	407 (49.8)
Collective housing	1 (0.1)
Other (not specified)	2 (0.2)
Housing tenure^4^, n (%)	
Owned	694 (85.7)
Rented	116 (14.3)
Geographical area of dwelling^5^, n (%)	
City area	491 (60.2)
Urban area	248 (30.4)
Rural area	76 (9.3)
Cohabitants^6^, n (%)	
Single household	213 (26.2)
Partner, spouse	572 (70.3)
Other adults	8 (1.0)
Children under 18	31 (3.8)
Children over 18	48 (5.9)

Note. ^1^4 missing. ^2^0 missing. ^3^3 missing. ^4^10 missing. ^5^5 missing. ^6^6 missing

### Data Collection

In phase 1 the user panel received the UJACAS, a response envelope, and a questionnaire based on the principles of CVI as described by Polit and colleagues (2007). The user panel was instructed to read through the items and use the questionnaire to write down any comments, and to rate each item on a scale from 1 to 4. A higher rating indicated understandability and relevance. Replies were sent back within two weeks.

The respondents in phases 2 and 3 received a postal letter with the UJACAS, and information to go through it and answer as much as possible before a telephone call to register their responses. For phase 2, respondents received two copies and were instructed to throw away the filled-out UJACAS immediately after the first telephone call (T1). A second telephone call was scheduled approximately two weeks later (T2), and the respondents were asked to fill out the second copy of UJACAS the day before. All data in phase 2 were collected by the first author using the same UJACAS version as the respondents. The mean time to complete the questionnaire via telephone was 22 min (range = 9–70 min; SD = 11 min) T1, and 13 min (range = 3–55 min; SD = 8.5 min) for T2.

In phase 3, data were collected by telephone calls made by two experienced occupational therapists, one of which being the first author collecting approximately 300 of the respondents’ answers. Also, two occupational therapy program students collected 200 of the answers. Prior to data collection, all staff involved received information about the instrument and the specific item meanings, also a written summary to use as a tutorial during the telephone calls. The students were offered regular guidance to deal with any issues that might arise during the data collection. The responses to UJACAS were documented directly into a web platform set up for Prospective RELOC-AGE.

### Assessing Semantic Equivalence and Content Validity (Phase 1)

Semantic equivalence was assessed after a professional translation of the Finnish original instrument to English and Swedish. To compare between languages the research team as described above, were involved in discussions about the phrasing and underlying meaning of the items in an iterative process. The instrument creator (TR) had some linguistic proficiency in Swedish and contributed to the comparison between languages and clarified the underlying ideas of the UJACAS. To further assess how the phrasing was perceived by the users, a user panel was instructed to rate each item for understandability (Is the item easy or hard to understand?) and relevance (Is the item relevant to describe active ageing?), and also write down any comments they had regarding specific items and the UJACAS in its entirety. Semantic equivalence was considered established when consensus was reached, after iterative discussions that were documented and analysed consecutively.

Based on the user panel ratings, content validity was evaluated using content validity index (CVI) for both individual items and total scale (Polit & Beck, 2006; Polit et al., 2007). For individual items a sum score of the ratings divided by the number of raters generated a mean score. Based on the mean score of items, scale CVI Average was calculated for the total scale. A higher CVI value indicated a higher rating of content validity. Responses in the comment fields were sorted by similarities and differences and used for fine-tuning of the phrasing of the individual items.

### Data Analyses Concerning Data Quality, Floor and Ceiling Effects, Test-Retest Reliability, Internal Consistency and Construct Validity (Phase 2 and 3)

Imputation for missing answers in the UJACAS was applied using the following formula: (sum score / sub-questions responded to) × sub-questions offered (Rantanen et al., 2019). Floor and ceiling effects were described on the subscale and total score levels using cut off vales 15–20% as described by Hobart and Cano (2009).

The test–retest reliability of the subscales and total scale was assessed using the intraclass correlation coefficient (ICC) (Koo & Li, 2016). Based on the 95% confident interval, ICC values were interpreted as poor (< 0.5), moderate (between 0.5 and 0.75), good (0.75–0.90), and excellent (> 0.90). ICC estimates and their 95% confidence intervals were calculated based on a single rater, absolute-agreement, 2-way mixed-effects model.

Internal consistency reliability was assessed using Cronbach’s alpha, interpreting values > 0.70 and corrected total-item correlation of the sub-questions > 0.3 (Hobart & Cano, 2009; Streiner et al., 2015) as acceptable. The standard error of measurement (SEM) was calculated and complemented with a 95% confidence interval (Hobart & Cano, 2009).

To evaluate convergent and discriminant aspects of construct validity, three hypotheses were pre-defined based on previous research (Baker et al., 2003; Bombak, 2013; Rantanen et al., 2019, 2021) and clinical reasoning. Addressing convergent validity, Pearson correlation was used (Streiner et al., 2015). We hypothesized that a higher level of active ageing was expected to correlate significantly with: (1) Self-Rated Health assessed with the 1-item question from the SF-12 scale (Jenkinson et al., 1997), “In general, would you say your health is…” (5 response options ranging from poor to excellent). (2) Life-Space Mobility Assessment, Swedish version (Fristedt et al., 2016) questions 4 and 5, whether respondents during the previous 4 weeks had been outside their neighbourhood, town, or beyond town. For each level, respondents indicated how often (< once per week; 1 to 3 times per week; 4 to 6 times per week; every day), and whether they needed a technical device/assistance (sum score = (4 × LS4 score) + (5 × LS5 score), ranging from 0 to 36). Higher scores indicated greater life-space mobility. Correlation values were considered as weak between 0.1 and 0.3; moderate between 0.4 and 0.6); strong between 0.7 and 0.9 and perfect at 1.0 (Akoglu, 2018). Addressing discriminant validity, Mann Whitney U test was used to differentiate UJACAS sum scores between persons who have had been diagnosed with clinical depression and those who had not, assessed with the question “Have you been diagnosed with clinical depression”, (Yes, during the last 12 months; Yes, but not during the last 12 months; No, never). As the risk of relapse of depression is high (Burcusa & Iacono, 2007; Luijendijk et al., 2008).

two groups were created, those who answered they had had depression at any point, and those who answered they had not. We hypothesized that a lower level of active ageing was expected among persons that had previously been diagnosed with clinical depression.

P-values < 0.05 were considered statistically significant. All statistical analyses were computed using IBM SPSS statistical package version 27.

**Table 2:** Analyses in the three study phases

Analysis	Phase no.		
	1^a^	2^b^	3^c^
Content validity (content validity index)	X		
Data quality		X	X
Floor and ceiling effects		X	X
Test-retest reliability (intraclass correlation coefficient)		X	
Internal consistency (Cronbach’s alpha and corrected total-item correlation)			X
Construct validity (convergent and discriminant hypothesis testing)			X

Note. ^a^Phase 1, Semantic equivalence including the research team and a user panel; ^b^Phase 2, test-retest; ^c^Phase 3, UJACAS included in Prospective RELOC-AGE

## Ethics

For phase 1, we involved older adults as experts rather than study participants, which does not require formal ethical approval. Following the principles of the Helsinki Declaration and current national legislation and policies on ethics for research involving humans, for phase 2 no sensitive personal data were collected. Accordingly, formal ethical approval according to current Swedish legislation did not apply. The participants’ delivery of a completed questionnaire counted as informed consent. Prospective RELOC-AGE was approved by the Swedish Ethical Review Authority (No. 2020-03457). All participants in Prospective RELOC-AGE gave their written informed consent. Participants in the study were given written and verbal information about the study, and they were informed of the possibility to opt out at any time without further consequences.

## Results

### Translation and Semantic Equivalence (Phase 1)

Descriptions of all 17 items and related sub-scale phrasings in the Swedish version delivered by the translator were fine-tuned to comply with the underlying meaning, and the linguistic expressions were optimised. For instance, Goals subscale of item Use computer/pad” originally read “I have wanted to use a computer or an iPad”. This item was discussed as to what purpose digital technology was used for, for example, the use of internet or writing down recipes, and why the item did not include hardware such as smartphones and surf tablets. After discussions the item was re-phrased to (Goals subscale) “I have wanted to use digital technology”, thus including a broader interest for a range of devices. All items were discussed, optimised, and fine-tuned in a similar process. Finally, comments from the user panel (see below) were considered, and the final version of UJACAS in Swedish was established.

### Content Validity (Phase 1)

Content validity was overall assessed as high. The user panel was positive to the Swedish version of the UJACAS regarding relevance as well as understandability in phrasing of most of the items. However, the items Public matters, and Faith or world view were rated lower regarding understandability as well as relevance, see Table 3. Comments suggested the phrasing of item Public matters, as “not Swedish”, and item Interesting days was described as making a judgement with the way it was expressed. Some suggested that item Faith or world view did not apply due to their agnostic world view. These items were fine-tuned and sent to the user panel a second time, after which the panel members approved the phrasing.

**Table 3:** Result of user panel evaluation of content validity; Content validity index (CVI)

	Understanding item phrasing							Relevance for active ageing											
User panel member no.	1	2	3	4	5	6	Item CVI	1		2		3		4		5		6	Item CVI
Abbreviated item title																			
1. Crafting & DIY	4	4	4	4	3	3	3.67	4		4		2		3		4		3	3.33
2. Artistic pursuits	4	3	4	4	4	3	3.67	4		4		3		4		4		3	3.67
3. Events	4	4	4	4	4	3	3.83	4		4		4		4		4		3	3.83
4. Nature	-	4	4	4	4	4	3.33	-		4		4		4		4		4	3.33
5. Physically fit	4	4	4	4	4	4	4.00	4		4		4		4		4		4	4.00
6. Exercise mind	4	2	4	3	4	3	3.33	4		4		4		4		4		3	3.83
7. Use computer/pad	4	4	4	4	4	3	3.83	4		4		4		4		4		2	3.67
8. Support others	4	3	4	4	4	3	3.67	4		4		4		4		2		3	3.50
9. Social relationships	4	4	4	4	4	4	4.00	4		4		4		4		3		4	3.83
10. New acquaintances	4	4	4	4	4	4	4.00	4		3		4		4		2		4	3.50
11. Promote own life	4	3	4	4	4	3	3.67	4		4		4		4		4		3	3.83
12. Public matters	4	2	1	4	2	2	2.50	4		3		1		4		2		2	2.17
13. Interesting days	4	2	4	4	2	4	3.33	4		4		4		4		3		4	3.83
14. Make home cosy	4	4	4	4	3	3	3.67	4		4		4		4		4		3	3.83
15. Appearance	4	4	4	4	4	3	3.83	4		4		4		4		3		3	3.67
16. Financial affairs	4	4	4	3	4	4	3.83	4		4		4		4		4		4	4.00
17. Faith and world view	4	2	1	4	2	3	2.17	4		4		-		4		2		3	2.83
Total	3.76	3.35	3.65	3.88	3.53	3.29		3.76		3.88		3.41		3.94		3.35		3.24	
Scale CVI Average							3.55												3.57

Note. Scale from 1 (hard/irrelevant) to 4 (easy/relevant); - indicates missing data

### Data Quality Including Floor- and Ceiling Effects (Phase 2 and 3)

For phase 2 and 3 data quality was overall high with no cases of missing data in phase 2, and 0.5% cases in phase 3, see Table 4. In phase 2 the ratings showed no apparent clustering towards minimum or maximum values except for in the subscale Ability. On T1 maximum subscale score was attained by 23 respondents (36.5%), and 21 respondents (33.3%) on T2. In phase 3, 177 (21.6%) persons registered maximum scores in the Ability subscale.

**Table 4:** Descriptive UJACAS data from phases 2 (*N* = 63) and 3 (*N* = 820), and indicators of data quality

	Mean score of ratings (SD) Range			Floor effect N (%)			Ceiling effect N (%)				Missing data M (%)			
UJACAS subscale	Phase 2		Phase 3	Phase 2		Phase 3	Phase 2			Phase 3	Phase 2		Phase 3	
	T1	T2		T1	T2		T1	T2			T1	T2		
Goals (17 items)	49.8 (7.4) 30–64	50.9 (7.5) 32–67	48.6 (8.5) 19–68	0	0	0	0	0		2 (0.2%)	0	0	1 (0.1%)	
Ability (17 items)	64.1 (5.0) 47–68	63.5 (5.8) 43–68	62.1 (6.4) 26–68	0	0	0	23 (36.5%)	21 (33.3%)		177 (21.6%)	0	0	1 (0.1%)	
Opportunity (17 items)	51.3 (7.3) 38–66	50.8 (8.7) 29–68	54.8 (9.5) 18–68	0	0	0	0	1 (1.6%)		48 (5.9%)	0	0	1 (0.1%)	
Activity (17 items)	44.9 (7.2) 30–59	44.8 (7.2) 40–49	42.7 (8.4) 13–67	0	0	0	0	0		0	0	0	4 (0.5%)	
Total score (68 items)	210.1 (19.0) 158–244	210.6 (19.8) 164–245	208.3 (24.9) 102–266										4 (0.5%)	

Note. Min-max for each subscale is 0–68, and for the total score 0-272; higher sum score indicates a higher level of active ageing

### Test-Retest Reliability (Phase 2)

On the total scale level, good test-retest reliability was indicated with a result of 0.88 (95% confidence interval 0.80–0.93). Subscales Goals, Ability and Activity achieved good reliability, whereas Opportunity reached a moderate level, see Table 5.

**Table 5:** Results regarding reliability and standard error of measurement, phases 2 and 3

UJACAS subscale	ICC (CI 95%)	Cronbach’s α	SEM (CI 95%)	CITC Median (range)
	Phase 2 (*N* = 63)	Phase 3 (*N* = 820)		
Goals	0.85 (0.76–0.91)	0.78	3.99 (40.79–56.42)	0.4 (0.19–0.49)
Ability	0.82 (0.71–0.90)	0.85	2.48 (57.24–66.96)	0.5 (0.22–0.60)
Opportunity	0.71 (0.53–0.83)	0.87	3.43 (48.09–61.51)	0.5 (0.27–0.66)
Activity	0.90 (0.83–0.94)	0.73	4.36 (34.15–51.26)	0.3 (0.03–0.48)
Total score	0.88 (0.80–0.93)	0.91	7.47 (193.66-222.94)	

Note. ICC = intra-class correlation; SEM = standard error of measurement; CITC = corrected item-total correlation

### Standard Error of Measurement (Phase 3)

On the total scale level, the analysis om SEM showed that 7.47 points indicate a real change, while the corresponding subscale points ranged from 2.48 to 4.36, see Table 5.

### Internal Consistency (Phase 3)

Addressing homogeneity, Cronbach´s alpha was high (0.91) on total scale level. Although lower on subscale level with values ranging from 0.73 to 0.87, all surpassed the cut-off of > 0.7.

Corrected total-item correlation showed that 57 sub-questions (84%) reached the cut-off value of 0.3. Regardless of what subscale, the item Use computer/pad ranged between 0.03 and 0.29, and the item Financial affairs (range = 0.20–0.29) did not surpass the cut off value of 0.3. For the subscale Activity, four items (Crafting & DIY, Events, Physically fit, Exercise mind) did not surpass the cut off, see Table 5.

### Construct Validity (Phase 3)

Assessed with UJACAS, active ageing was significantly correlated with self-rated health (*r* = 0.41; p-value < 0.01) as well as with life-space mobility (0.24; p-value < 0.01). A slightly lower level of active ageing was found among persons who had been diagnosed with clinical depression (Median = 209.0, *N* = 108), compared to those who had not (Median = 211.0, *n* = 705, *r* = 0.07; *p* = 0.052).

## Discussion

In the present study, a new self-assessment to capture active ageing on the individual level – UJACAS (Rantanen et al., 2019) - was translated to Swedish and evaluated for psychometric properties. Semantic equivalence was established prior to evaluating reliability and validity in a Swedish context involving people aged 55+. With three phases, the different psychometric aspects could be assessed in three sample groups, using a larger sample concluding the analyses. Including participants representing a relevant population segment for self-assessment of active ageing, the Swedish version of the UJACAS generates data of high quality with no floor effects and acceptable ceiling effects on the total scale level. In accordance with previous studies, test-retest reliability (Rantanen et al., 2019) and internal consistency (Erbil & Hazer, 2019) were acceptable, and homogeneity results indicate that the items can be summed to a total score (Hobart & Cano, 2009). The SEM result indicate that a change in UJACAS total scores should exceed 7.47 points to indicate a real change (Hobart & Cano, 2009), which is important for future research using active ageing as an outcome measure. Like in the study in Finland where UJACAS was originally developed (Rantanen et al., 2019), construct validity in relation to a set of relevant concepts was established, confirming a priori stated hypotheses. Further research of the relationship between depression and active aging is warranted due to the small difference found regarding clinical depression and, also the potential biased in sub-scale activity due to the COVID-19 pandemic as described in previous research (Zingmark et al., 2022).

Examining the scores for different data collection occasions, test-retest values indicated an overall good test-retest reliability. These findings are in line with similar evaluation performed during the development of the instrument (Rantanen et al., 2019). Although not exactly comparable due to methodological differences, our findings are in line with the results regarding a Turkish version of the UJACAS where a positive correlation was found (Erbil & Hazer, 2019).

Alpha values and corrected total-item correlation on total level indicate that the four subscales measure the same underlying concept (Mokkink et al., 2019). In previous studies the total scale alpha levels were higher, that is, alpha = 0.95 (Rantanen et al., 2019) and 0.97 (Erbil & Hazer, 2019). Still, while lower in our study for subscales as well as for the total scale, the results surpassed the accepted level of 0.7. Actually, according to Taber (2018) a very high alpha levels indicate a problem in terms of item redundancy, which was not the case in the present study. It should be noted that consistently across studies the subscale alpha levels were lower than for the total scale (Erbil & Hazer, 2019; Rantanen et al., 2019), indicating that the UJACAS should be used in its entirety (Mokkink et al., 2019).

While corrected total-item correlation showed that 84% of the sub-questions reached the cut-off value of 0.3, two items (i.e., Use computer/pad and Financial affairs) did not for any of the four subscales. This might indicate that those items do not measure the same underlying property (Hobart & Cano, 2009) as the rest of the UJACAS. Moreover, within the Activity subscale four sub-questions (i.e., Crafting & DIY; Events; Physically fit; Exercising mind) failed to reach the cut-off value. In the light of our experiences during the data collection, where items such as Events, Physically fit, Social relationships and New acquaintances stood out as being commented a lot by the respondents, the Activity subscale might have been affected by the COVID-19 pandemic (Zingmark et al., 2022). The comments described how the perception of such items had changed because of the national pandemic restrictions particularly for persons 70+ (The Public Health Agency of Sweden, 2020). The respondents described that their circumstances and possibilities regarding activities had changed, as had their ability and will to act to some extent. However, one study (Kivi et al., 2020) reported that Swedish people in their 60s remained stable in terms of life satisfaction and loneliness, and that self-rated health and financial satisfaction slightly improved in the early stage of the pandemic. On the other hand, Rantanen et al. (2021) found that life-space mobility, active ageing and quality of life decline coincided, and that less decline in quality of life was accompanied by a smaller decline in active ageing scores (Rantanen et al., 2021). Furthermore, in Finland the number of activity destinations were reported as reduced by half as a result of social distancing recommendations (Portegijs et al., 2021). Thus, it is reasonable to assume that activity levels were skewed during the time of data collection due to the COVID-19 pandemic (Zingmark et al., 2022). Accordingly, forthcoming studies from Prospective RELOC-AGE, based on data during as well as after the pandemic, have potential to further elucidate these findings.

Our results indicate that the construct validity of the Swedish version of UJACAS is strong, with low to medium correlations (Akoglu, 2018) showing that UJACAS is related to with the associated measures, although different. These results are consistent with previous research (Rantanen et al., 2019) showing associations between active ageing, self-rated health, and life-space mobility. Life-space mobility is often associated with independence (Fristedt et al., 2016), and self-rated health using the SF-12 scale provides information on physical and mental health (Jenkinson et al., 1997). Our result regarding discriminant validity revealed a small difference in median-value that could be interpreted as an indication of an association between active ageing and depression. Depression has been described as one of the most common mental illnesses (Burcusa & Iacono, 2007) and varies from just under 1 to 29% in the population in Sweden 60+ (Horackova et al., 2019; Karlsson et al., 2016; Public Health Agency of Sweden, 2019; Wiberg et al., 2013). According to WHO, depression is common and underdiagnosed in the oldest old, and that untreated depression can lead to poor health and increased mortality (World Health Organisation, 2021), and affects a person in different ways; chronic illness, pain, limitations in activities of daily living tools, grip strength, and cognitive impairment (Horackova et al., 2019). Since potentially biased results in activity levels (Zingmark et al., 2022) may have influenced the assessment of discriminant validity in this study, further research of the relationship between depression and active aging is warranted.

### Strengths and Limitations

To strengthen the methodological aspects of the evaluation of test-retest reliability (phase 2) (Mokkink et al., 2019), all data was collected by the first author. Also, a duration of 2 weeks in between calls and instructing the respondents to throw away the used questionnaire directly after the first call were to ensure the answers were unaffected.

This study may be vulnerable to bias. In phase 2, 13 senior citizen associations were contacted to invite persons to participate. At that time, all associations were required to follow national pandemic restrictions, and thus contact with members was primarily made digitally. This makes it probable that there were persons who never received the information and thus not the opportunity to participate, creating a pre-screening bias (Poli et al., 2021). For phases 2 and 3 convenience sampling was used, risking a volunteer bias (Boughner, 2012). Although such sampling often is used, the generalisability of the results is questionable (Hultsch et al., 2016). To reduce such risk, we used different recruitment strategies, applied strategies to increase the proportion of volunteers, and to ensure confidentiality (Brassey et al., 2017). Still, it is likely that those who participated were more digitally literate than the general population of people aged 55+. The proportion of non-users of the Internet is declining in Sweden but still represents every fifth pensioner (65+) (The Swedish Internet Foundation, 2021). The older age, the higher the proportion of non-users. In addition, respondents had a higher education level than the general population over 55 + (17%) (Statistics Sweden). The participants in phase 2 received information that their individual answers were confidential and would only be reported on group level, and no sensitive personal information was collected. Phase 3 respondents received a computer-generated, individual code to log in directly to the database of the survey. Although some questions in the telephone interview might have been considered as sensitive, the respondents received the questions beforehand along with instructions to read through them to prepare. Thus, the questions were known to the phase 2 and 3 respondents, and they were informed that they could skip a question at any time or opt out from the interview without any further consequences. However, all phase 2 respondents completed the test-retest on both occasions with high data quality, and only two chose to end their participation before the first call. In phase 3, 1,964 persons answered the web survey and 1,412 of those also wanted to participate in the telephone interview. Of the 1,011 invited to the telephone interview, 820 completed. This suggests that the respondents considered the relevance of the project and indicates that they represent a relevant target population for the present study (Brassey et al., 2017).

## Conclusion

This study suggests that the Swedish version of UJACAS is reliable and valid to capture active ageing on the individual level among persons aged 55 + in Sweden. With the systematic measurement error reported real changes can be detected, making UJACAS suitable for use in longitudinal studies as well as in intervention research using active ageing as an outcome. The results should be interpreted with the ongoing COVID-19 pandemic in mind, and more knowledge is needed regarding whether and how this situation affects active ageing.

## Data Availability

Data will be available on reasonable request. Please contact susanne.iwarsson@med.lu.se for more information.
